# Possible association between dysnatremias and mortality during hospitalization in patients undergoing acute hemodialysis: analysis from a Peruvian retrospective cohort

**DOI:** 10.1590/2175-8239-JBN-2018-0243

**Published:** 2019-09-12

**Authors:** Edward Mezones-Holguin, Roberto Niño-Garcia, Percy Herrera-Añazco, Álvaro Taype-Rondan, Josmel Pacheco-Mendoza, Adrian V. Hernandez

**Affiliations:** 1 Universidad San Ignacio de Loyola, Vicerrectorado de Investigación, Lima, Peru.; 2 Epi-gnosis Solutions. Piura, Peru.; 3 Universidad Nacional de Piura, Facultad de Ciencias de la Salud, Sociedad Científica de Estudiantes de Medicina de la Universidad Nacional de Piura (SOCIEMUNP), Piura, Peru.; 4 Hospital Nacional 2 de mayo, Department of Nephrology, Lima, Peru.; 5 University of Connecticut/Hartford Hospital Evidence-based Practice Center, Hartford, CT, USA.

**Keywords:** Acute renal injury, Renal Insufficiency, Chronic, Dialysis, Chlorine, Sodium, Mortality, Lesão Renal Aguda, Insuficiência Renal Crônica, Diálise, Cloro, Sódio, Mortalidade

## Abstract

**Objective:**

To evaluate the association between dysnatremias or dyschloremias and mortality during hospitalization in patients with acute kidney injury (AKI) or chronic kidney disease (CKD) undergoing acute hemodialysis.

**Methods:**

We carried out a retrospective cohort study on adult patients undergoing acute hemodialysis with AKI or CKD diagnosis at a public hospital in Lima, Peru. Dysnatremias were categorized as hyponatremia (Na < 135mmol/L) or hypernatremia (Na > 145mmol/L), and dyschloremias were defined as hypochloremia (Cl < 98 mmol/L) or hyperchloremia (Cl > 109mmol/L). The outcome of interest was mortality during hospitalization. We performed generalized lineal Poisson family models with bias-corrected and accelerated non-parametric bootstrap to estimate the risk ratios at crude (RR) and adjusted analysis (aRR) by gender, age, HCO3 (for all patients) and Liaño score (only for AKI) with CI95%.

**Results:**

We included 263 patients (mean age: 54.3 years, females: 43%): 191 with CKD and 72 with AKI. Mortality was higher in patients with AKI (59.7%) than in patients with CKD (14.1%). In overall, patients with hypernatremia had a higher mortality during hospitalization compared to those who had normal sodium values (aRR: 1.82, 95% CI: 1.17-2.83); patients with hyponatremia did not have different mortality (aRR: 0.19, 95% CI: 0.69-2.04). We also found that hyperchloremia (aRR: 1.35, 95% CI: 0.83-2.18) or hypochloremia (aRR: 0.66, 95% CI: 0.30-14.78) did not increase mortality in comparison to normal chloride values. No association between dysnatremias or dyschloremias and mortality during hospitalization was found in CKD and AKI subgroups.

**Conclusions:**

In our exploratory analysis, only hypernatremia was associated with mortality during hospitalization among patients with AKI or CKD undergoing acute hemodialysis.

## Resumo

**Objetivo:**

Avaliar a associação entre distúrbios do sódio ou do cloro e mortalidade hospitalar de pacientes com insuficiência renal aguda (IRA) ou doença renal crônica (DRC) submetidos a hemodiálise aguda.

**Métodos:**

O presente estudo de coorte retrospectiva incluiu pacientes adultos submetidos a hemodiálise aguda com diagnóstico de IRA ou DRC em um hospital público de Lima, Peru. Os distúrbios do sódio foram classificados como hiponatremia (Na < 135mmol/L) ou hipernatremia (Na > 145mmol/L), enquanto os distúrbios do cloro foram classificados como hipocloremia (Cl < 98 mmol/L) ou hipercloremia (Cl > 109mmol/L). O desfecho de interesse foi mortalidade hospitalar. Utilizamos modelos de Poisson da família de modelos lineares generalizados com bootstrap não-paramétrico e correção de viés acelerado para estimar os riscos relativos na análise bruta (RR) e ajustada (RRa) para sexo, idade, HCO3 (para todos os pacientes) e escore de Liaño (apenas para IRA) com IC 95%.

**Resultados:**

Foram incluídos 263 pacientes (idade média 54,3 anos; 43% do sexo feminino), 191 com DRC e 72 com IRA. A mortalidade foi mais elevada nos pacientes com IRA (59,7%) do que nos indivíduos com DRC (14,1%). No geral, os pacientes com hipernatremia tiveram mortalidade hospitalar mais elevada do que os indivíduos com valores normais de sódio (RRa: 1,82; IC 95%: 1,17-2,83). Os pacientes com hiponatremia não apresentaram mortalidade diferente (RRa: 0,19; IC 95%: 0,69-2,04). Também identificamos que hipercloremia (RRa: 1,35; IC 95%: 0,83-2,18) e hipocloremia (RRa: 0,66; IC 95%: 0,30-14,78) não elevaram a mortalidade em comparação a indivíduos com níveis normais de cloro. Não foi encontrada associação entre distúrbios do sódio ou do cloro e mortalidade hospitalar nos subgrupos com DRC e IRA.

**Conclusões:**

Em nossa análise exploratória, apenas hipernatremia apresentou associação com mortalidade hospitalar em pacientes com IRA ou DRC submetidos a hemodiálise aguda.

## Introduction

Electrolytes and their alterations have an important repercussion in health, and it supposed to be an important challenge in clinical practice. These molecules contribute to maintaining human body homeostasis^1^. Chloride (Cl) and sodium (Na) and their alterations - called dyschloremias and dysnatremias, respectively - have been associated with several harmful events in acute and chronic clinical situations^2^^-^ 12. Patients with kidney diseases are a special population of interest.

These patients are more likely to have alterations of Na and Cl, and to produce deleterious effects on their health. These include changes of urine dilution and concentration in response to antidiuretic hormone, and alteration of water and Na and CI reabsorption and excretion^13^^,^ 14. Previous studies have reported that dysnatremias and hypochloremia are associated factors for mortality in patients with chronic kidney disease (CKD), with or without dialysis^15^^-^ 22. Thus, dyschloremias and dysnatremias have been proposed as potential useful factors for mortality prediction in this population^15^^-^ 22.

Dysnatremias and dyschloremias in patients with acute kidney injury (AKI) or CKD submitted to acute dialysis need special attention. It is plausible that in AKI patients there is an association between these alterations and mortality. However, there are a few studies reporting the associations between hyperchloremia^13^ or dysnatremias with mortality in AKI patients undergoing hemodialysis^23^. Likewise, we have not found publications about the association between Na and Cl alterations and mortality in patients with CKD undergoing acute hemodialysis.

Our study evaluated the association between dysnatremias or dyschloremias with hospital mortality in patients with AKI or CKD undergoing acute hemodialysis. Findings could be useful for the improvement of clinical management in these patients.

## Methods

### Design and participants

We carried out a retrospective cohort in adult patients with a diagnosis of AKI or CKD undergoing acute hemodialysis at the Nephrology Department of National Hospital 2 de Mayo, a public general hospital of Ministry of Health located in Lima, Peru, between January 2015 and July 2017.

AKI or CKD diagnosis, as well as the indication for hemodialysis, were determined by an attending nephrologist based on clinical criteria proposed in the Kidney Disease Improving Global Outcomes (KDIGO) guidelines^24^. We excluded patients admitted to dialysis without kidney disease (vg. methanol intoxication, etc.) and patients who had received dialysis in other hospitals (since we could not access to the laboratory results and clinical evaluation before initiating the hemodialysis). Acute hemodialysis was defined as the emergency dialysis in a patient who never have received dialysis before. Both groups of patients (AKI and CKD) had conventional hemodialysis. In average, they had three sessions per week with a duration of 3.5-4 hours, using low-flow biocompatible synthetic membranes (polysulfone).

### Exposure, outcome, and other patient variables

Dysnatremias and dyschloremias were the exposure variables. Electrolytes were measured using a dry chemical technique in the last sample obtained in 24 hours before starting hemodialysis. Based on NA serum values, dysnatremias were categorized as hyponatremia (lower than 135 mmol/L) and hypernatremia (higher than 145 mmol/L). We also classified Dyschloremia in the function of Cl serum values in two categories: hypochloremia (lower than 98 mmol/L) or hyperchloremia (higher than 109 mmol/L). Our outcome was mortality and it was defined as death during hospitalization.

We assessed age and sex as demographic variables. Also, we measured serum potassium (K, categorized in: < 3.5 mmol/L, 3.5 mmol/L to 5.5 mmol/L, and > 5.5 mmol/L) and bicarbonate (HCO3, categorized in: 24 mmol/L, 24 mmol/L to 25 mmol/L and > 25 mmol/L) within 24 hours before starting dialysis. The Anion Gap (AG) value was calculated by the formula: Na - (Cl + HCO3) and categorized as high (higher than 12) and normal (lower than 12). The ratio between AG delta (12 - AG from patients) and bicarbonate delta (24 - bicarbonate from patients) was used to define the triple disorder. A ratio < 1 implied coexistence of high AG metabolic acidosis with normal AG, and ratio > 2 defined the coexistence of high AG metabolic acidosis and metabolic alkalosis (AM).

In patients with AKI, we assessed the severity of their clinical condition using the Liaño severity index according the equation:^25^

$$\begin{array}{c} \mathrm{Severity}=0.032^{*}\left(\mathrm{age}\;\mathrm{in}\;\mathrm{decades}\right)-\\ 0.086^{*}\left(\mathrm{male}\;\mathrm{sex}\right)-0.109^{*}\left(\mathrm{nephorotoxic}\right)+0.109^{*}\\ \left(\mathrm{oliguria}\right)+0.116^{*}\left(\mathrm{hypotension}\right)+0.122^{*}\left(\mathrm{jaundice}\right)\\ +0.15\left(\mathrm{coma}\right)-0.154^{*}\left(\mathrm{normal}\;\mathrm{consciousness}\right)+\\ 0.182^{*}\left(\mathrm{assisted}\;\mathrm{respiration}\right)+0.21. \end{array}$$

We used the recommended ≥ 0.74 threshold based on the association with a higher mortality in a Peruvian cohort^25^. All variables were obtained from the epidemiological surveillance records of the Nephrology Department.

### Statistical analyses

We used arithmetic means and standard deviations (SD), and frequencies and percentages to report numeric and categorical variables, respectively. When evaluating the best multiple regression model for current data, none of them fulfilled assumptions. Also, there were fewer observations in certain categories of variables. In view of this, we replaced the estimation of the association measures and their uncertainty by non-parametric bootstrap, a resampling technique that does not require the development and fulfillment of assumptions about the probabilistic structure of observations. To estimate the confidence intervals, 1000 replicates were performed with the bias-corrected and accelerated method for generalized linear models of the Poisson family^26^. We estimated crude and adjusted risk ratios (RR and aRR) with 95% confidence intervals (CI) as association measure between exposure and outcome in all patients, and in AKI and CKD populations. Other variables were included in adjusted models based on epidemiological criteria (theoretical confounding factor) with measured variables^27^. Therefore, for all patients and CKD patients we adjusted for gender, age and HCO3 and in AKI patients, we adjusted for gender, age, HCO3 and Liaño score ≥ 0.74.

### Ethical considerations

This study is an analysis of epidemiological surveillance without sensitive patient information. The protocol was approved for the Institutional Research and Training department of Hospital Nacional 2 de Mayo from Lima, Perú.

## Results

From 275 patients with diagnosis of AKI or CKD admitted due to acute hemodialysis, we excluded 12 of them since they did not have sodium or chloride measurements. Finally, we included 263 patients: 72 with AKI and 191 with CKD.

**Figure 1:**
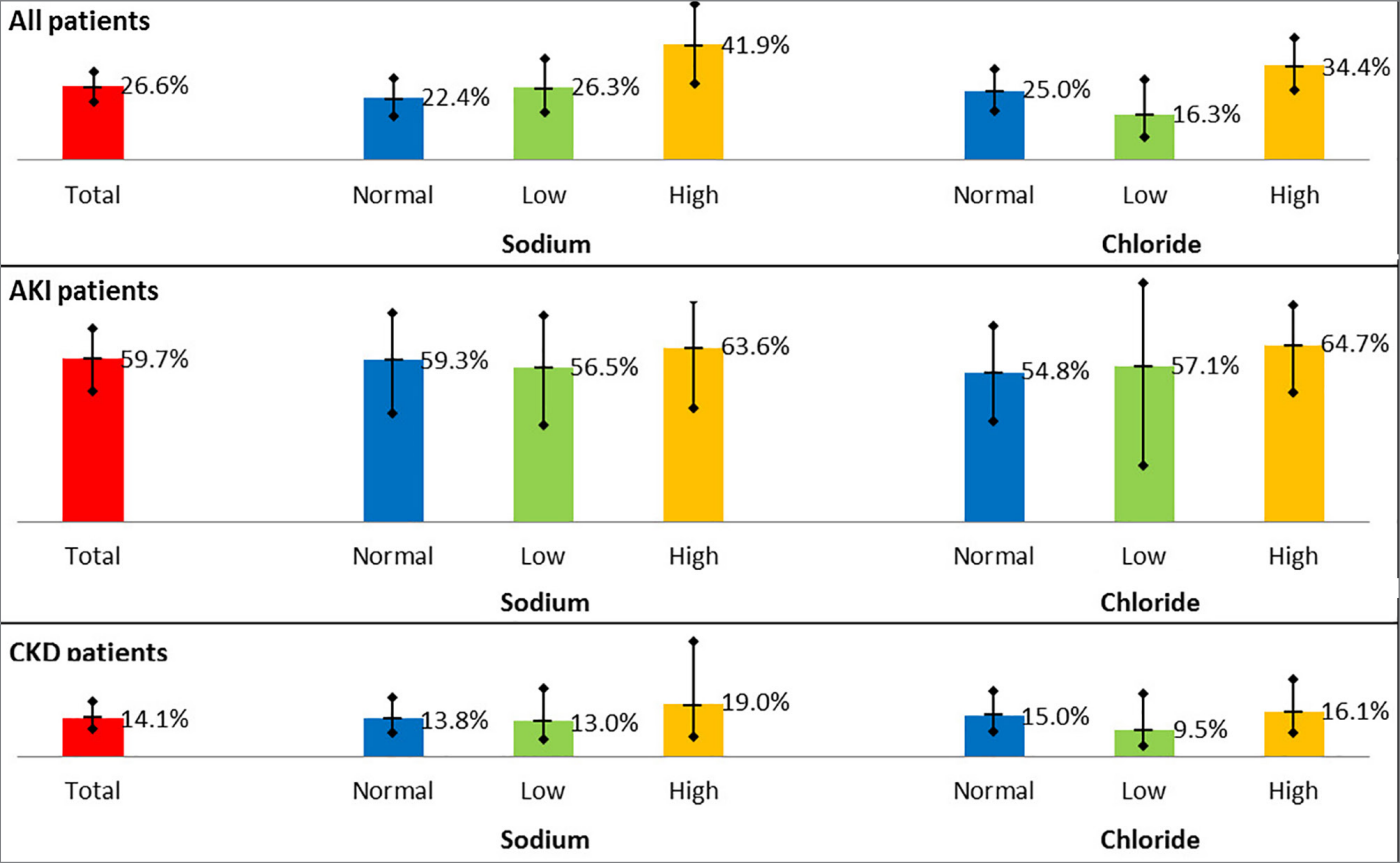
Distribution of mortality according Sodium and Chrolide levels in Acute Kidney Injury, Chronic Kidney Disease and all patients.

The average age was 54.7 (± 17.6) years and 54.3 (± 15.3) in patients with AKI and CKD, respectively. Most patients were men (57%). The average sodium values were 140.1 (± 10.4) mmol/L and 137.8 (± 7.3) mmol/L in patients with AKI and CKD, respectively (*p =* 0.049). Low sodium levels (< 135 mmol/L) were more likely to occur in AKI (31.9%) than in CKD (28.3%) groups, whether hypernatremia (> 145 mmol/L) was more frequent in patients with AKI (30.6%) than in patients with CKD (11.0%) (*p* < 0.001). Average chloride values were 109.0 (± 9.5) mmol/L and 104.4 (± 8.9) mmol/L in patients with AKI and CKD, respectively (*p <* 0.001). Hypochloremia (< 98 mmol/L) was less frequent in patients with AKI (9.7%) than in patients with CKD (22.0%); in contrast, hyperchloremia (> 109 mmol/L) was more frequent in patients with AKI (47.2%) than in patients with CKD (29.3%) (*p =* 0.009). Average potassium values were 5.1 (± 1.2) mmol/L and 5.4 (± 1.2) mmol/L in patients with AKI and CKD, respectively (*p =* 0.025). The average anion gap values were 18.2 (± 7.1) mmol/L and 20.7 (± 6.5) mmol/L in patients with AKI and CKD, respectively (*p =* 0.001). Mortality during hospitalization was higher in AKI (59.7%) than CKD (14.1%) patients (*p <* 0.001). In Table 1, we showed demographic and clinical characteristics of the entire population, AKI and CKD populations. In Figure 1 we compared mortality among groups, according to chloride and sodium levels. We reported frequencies with a confidence interval to 95%.

**Table 1:** Demographic and clinical characteristics of the total, acute kidney injury and chronic kidney disease populations

Variable	Total Patients	AKI patients	CKD patients	*p* value*
	N = 263	N = 72	N = 191	
Age (mean ± SD)	54.4 ± 15.9	54.7 ± 17.6	54.3 ± 15.3	0.890
Sex, Female, n (%)	112 (42.6)	33 (45.8)	79 (41.36)	0.513
Sodium (mmol/L) (mean ± SD)	138.5 ± 8.3	140.1 ± 10.4	137.8 ± 7.3	**0.049**
Normal (135-145), n (%)	143 (54.4)	27 (37.5)	116 (60.7)	**< 0.001**
Low (< 135), n (%)	77 (28.3)	23 (31.9)	54 (28.3)	
High (> 145), n (%)	43 (16.4)	22 (30.6)	21 (11.0)	
Chloride (mmol/L) (mean ± SD)	105.6 ± 9.3	109.0 ± 9.5	104.4 ± 8.9	**< 0.001**
Normal (98-109), n (%)	124 (47.2)	31 (43.1)	93 (48.7)	**0.009**
Low (< 98), n (%)	49 (18.6)	7 (9.7)	42 (22.0)	
High (> 109), n (%)	90 (34.2)	34 (47.2)	56 (29.3)	
Potassium (mmol/L) (mean ± SD)	5.3 ± 1.2	5.1 ± 1.2	5.4 ± 1.2	**0.025**
Normal (3.5-5.5), n (%)	151 (57.4)	48 (66.6)	103 (53.9)	0.124
Low (< 3.5), n (%)	9 (3.4)	3 (4.2)	6 (3.2)	
High (> 5.5), n (%)	103 (39.2)	21 (29.2)	82 (42.9)	
HCO3 (mmol/L) (mean ± SD)^†^	13.2 ± 4.7	13.4 ± 4.9	13.1 ± 4.7	0.694
Normal (24 - 25)	3(1.2)	0 (0.0)	3 (1.7)	0.371
Low (< 24)	245 (98.0)	70 (100.0)	175 (97.2)	
High (> 25)	2 (0.8)	0 (0.0)	2 (1.1)	
pH (mean ± SD) ^†^	7.2 ± 0.5	7.1 ± 0.9	7.2 ± 0.2	0.116
Normal (7.4-7.5), n (%)	24 (9.6)	9 (12.9)	15 (8.3)	0.276
Low (<7.4), n (%)	226 (90.4)	61 (87.1)	165 (91.7)	
Anion Gap ‡	20.0 ± 6.8	18.2 ± 7.1	20.7 ± 6.5	**0.001**
< 8	7 (2.9)	3 (4.4)	4 (2.3)	0.056
8 to 12	23 (9.4)	11 (15.9)	12 (6.9)	
> 12	214 (87.7)	55 (79.7)	159 (90.8)	
Liaño score (≥ 0.74), n (%) ^¶^	NA	35 (48.6)	NA	NA
Mortality, Yes, n (%)	70 (26.6)	43 (59.7)	27 (14.1)	**< 0.001**

Note: SD: standard deviation; NA: Not applicable; AKI: Acute Kidney Injury; CKD: Chronic Kidney Disease.

† n = 264;

‡ n = 244;

¶ n = 72

* Estimated value is for the comparison between AKI and CKD patients.

When evaluating the association between dysnatremias or dyschloremias and mortality during hospitalization, crude and adjusted models showed differences in mortality among patients who had hypernatremia (aRR: 1.82, 95% CI: 1.17-2.83), but not in those with hyponatremia (aIRR: 1.19, 95% CI: 0.69 - 2.04) when compared to those who had normal sodium values. We found no differences in mortality during hospitalization among patients who had hyperchloremia (aRR: 1.35, 95% CI: 0.83-2.18) or hypochloremia (aRR: 0.66, 95% CI: 0.30-14.78) when compared to those who had normal chloride values. There was no association in the subgroups of AKI and CKD separately, as shown in Table 2.

**Table 2:** Relationship between chloride or sodium levels, with mortality in AKI and CKD patients (Non-parametric BCA boostrap)

Variable	Died n (%)		Crude RR	Adjusted RR
All	Yes (n = 70)	No (n = 193)	n = 263	n = 251*
Sodium				
Normal (135-145 mmol/L), n (%)	32 (22.4)	111 (77.6)	Ref	Ref
Low (< 135 mmol/L), n (%)	20 (26.3)	57 (74.0)	1.16 (0.67 - 1.99)	1.19 (0.69 - 2.04)
High (> 145 mmol/L), n (%)	18 (41.9)	25 (58.1)	**1.87 (1.15 - 3.05)**	**1.82 (1.17 - 2.83)**
Chloride				
Normal (98- 109 mmol/L), n (%)	31 (25.0)	93 (75.0)	Ref	Ref
Low (< 98 mmol/L), n (%)	8 (16.3)	41 (83.7)	0.65 (0.30 - 1.42)	0.66 (0.30 - 14.78)
High (> 109 mmol/L), n (%)	31 (34.4)	59 (65.6)	1.38 (0.89 - 2.12)	1.35 (0.83 - 2.18)
CKD patients	Yes (n = 27)	No (n = 164)	n = 191	n = 180**
Sodium				
Normal (135 - 145 mmol/L), n (%)	16 (13.8)	100 (86.2)	Ref	Ref
Low (< 135 mmol/L), n (%)	7 (13.0)	47 (87.0)	0.94 (0.37 - 2.37)	0.93 (0.34 - 2.59)
High (> 145 mmol/L), n (%)	4 (19.0)	17 (81.0)	1.38 (0.00 - 2560.93)	1.61 (0.73 - 35.42)
Chloride				
Normal (98 - 109 mmol/L), n (%)	14 (15.0)	79 (85.0)	Ref	Ref
Low (< 98 mmol/L), n (%)	4 (9.5)	38 (90.5)	0.63 (0.03 - 14.53)	0.66 (0.02 - 21.22)
High (> 109 mmol/L), n (%)	9 (16.1)	47 (83.9)	1.07 (0.47 - 2.42)	1.14 (0.48 - 2.66)
AKI patients	Yes (n = 43)	No (n = 29)	n = 72	n = 71***
Sodium				
Normal (135 - 145 mmol/L), n (%)	16 (59.3)	11 (40.7)	Ref	Ref
Low (< 135 mmol/L), n (%)	13 (56.5)	10 (43.5)	0.95 (0.58 - 1.58)	1.01 (0.59 - 1.71)
High (> 145 mmol/L), n (%)	14 (63.6)	8 (36.4)	1.07 (0.67 - 1.72)	1.00 (0.66 - 1.52)
Chloride				
Normal (98-109 mmol/L), n (%)	17 (54.8)	14 (45.2)	Ref	Ref
Low (< 98 mmol/L), n (%)	4 (57.1)	3 (42.9)	1.04 (0.05 - 22.88)	1.03 (0.51 - 2.05)
High (> 109 mmol/L), n (%)	22 (64.7)	12 (35.3)	1.18 (0.78 - 1.78)	1.12 (0.70 - 1.80)

* Adjusted for gender, age, HCO3.

** Adjusted for gender, age and HCO3.

*** Adjusted for gender, age, HCO3 and Liaño score ≥ 0.74.

## Discussion

Our findings showed that in a single general hospital in Peru there was an association between hypernatremia and mortality during the hospitalization of patients with AKI or CKD undergoing acute hemodialysis; nevertheless, there was no association in each group separately. Also, we found no association between dyschloremias and mortality in these patients. Our analysis represents one of the first published series regarding this topic in a developing country.

The pathophysiology of the association between dysnatremias and mortality is limited and inconclusive. Although dysnatremias can be associated with brain alterations, which can explain their association with mortality in critical patients^12^^,^ 28 ^,^ 29, it has not been determined that this mortality is due to dysnatremia or to an underlying disease^9^. In AKI patients in hemodialysis, only one study found an association between hypernatremia and mortality^23^, and hypernatremia was defined as serum Na > 156 mmol/L^23^. A study in the USA found an association between serum sodium level and mortality in patients with CKD on hemodialysis for sodium values < 138 mmol/L and ≥ 140 mmol/L, with a stronger association for values lower than 138 mmol/L^17^. This means that the association with mortality occurs at the extremes of sodium values^17^. To our knowledge, the current investigation represents a first report of the positive correlation between dysnatremia and mortality in patients in hemodialysis when including both groups (AKI and CKD).

The pathophysiological mechanism about the association between dyschloremias and mortality is complex and not completely explained. In the case of hypochloremia, it is possibly associated with an abnormal neutrophil function^30^^,^ 31; hyperchloremic metabolic acidosis (normal AG) increases the pro-inflammatory response, with increased levels of nitric oxide and interleukin (IL) -6, - 10 or tumor necrosis factor (TNF)^32^^,^ 33. However, not all the studies have found an association between dyschloremia and mortality^35^, possibly due to differences of included patients. Studies that found an association between hyperchloremia and mortality were frequently conducted in patients with higher Cl load due to the use of saline solution (0.9% NaCl)^2^^,^ 4. Likewise, 0.9% NaCl use is associated with mortality in some studies in surgical patients^34^^,^ 35. In our study, we could not determine the frequency of the use of 0.9% NaCl and the Cl load, and our patients with hyperchloremia did not have coexistence of normal AG metabolic acidosis. This could explain why we did not find an association between hyperchloremia and mortality contrary to other studies^36^^,^ 37.

Some studies in critical patients without AKI have suggested that the association between hyperchloremia and mortality only happens if the CI collection was conducted 72 hours after admission 4, a situation different of our study, where Cl was measured on the first day of hospitalization. It is possible that the association between dyschloremia and mortality in a patient submitted to acute hemodialysis is not independent of confounders such as comorbidities and the critical condition^5^. In our study, among AKI patients, the results did not vary when adjusting the analysis according to the level of severity as per the Liaño score.

Our study has some limitations. First, we could not objectively evaluate the level of severity, cause of hospitalization, comorbidities and other clinical condition of patients undergoing acute hemodialysis, which may be a confounding factor for our associations of interest. However, we defined our analysis as exploratory, thus our exposures have been cataloged as markers of a possible association with mortality. Second, we could not measure some factors that can affect the CI and Na values in each patient before initiating hemodialysis, such as the use of 0.9% NaCl, other solutions for parenteral hydration, diuretics and the state of blood volume or nutrition of the patient, among others. Third, the extrapolation of our results must be cautiously conducted, since protocols and clinical condition can differ from other centers for patients requiring acute hemodialysis. Fourth, this was not a representative AKI/CKD population, which could affect the external validity of the results. However, the main objective was to evaluate the existence of an association, and in this regard, it is considered that this can be done in non-representative populations^38^. Fifth, statistical power problems (the sample size is relatively small) may have happened especially when evaluating the association between hypernatremia in each group (AKI or CKD); and in the absence of a known population distribution it was decided to make nonparametric bootstrap^26^.

We suggest carrying out new studies that evaluate the causal effect of sodium and chlorine alterations in the mortality of patients with AKI and CKD, which include the measurement of other potentially confusing clinical variables with larger sample size.

## Conclusion

In this single-center cohort, there was an association between hypernatremia and mortality during the hospitalization of patients in acute hemodialysis; nevertheless, no association was found with dyschloremias and mortality. In addition, no association was found in AKI or CKD populations separately. This information is relevant primary evidence that can inform the decision-making in these populations. Larger observational, prospective studies are necessary to confirm these findings.

## References

[1] Guyton AC, Hall JE (2006). Tratado de Fisiología Médica.

[2] Thongprayoon C, Cheungpasitporn W, Cheng Z, Qian Q (2017). Chloride alterations in hospitalized patients: Prevalence and outcome significance. PLoS One.

[3] Boniatti MM, Cardoso PRC, Castilho RK, Vieira SR (2011). Is hyperchloremia associated with mortality in critically ill patients? A prospective cohort study. J Crit Care.

[4] Neyra JA, Canepa-Escaro F, Li X, Manllo J, Adams-Huet B, Yee J (2015). Association of Hyperchloremia With Hospital Mortality in Critically Ill Septic Patients. Crit Care Med.

[5] Tani M, Morimatsu H, Takatsu F, Morita K (2012). The incidence and prognostic value of hypochloremia in critically ill patients. ScientificWorld Journal.

[6] McCluskey SA, Karkouti K, Wijeysundera D, Minkovich L, Tait G, Beattie WS (2013). Hyperchloremia after noncardiac surgery is independently associated with increased morbidity and mortality: a propensity-matched cohort study. Anesth Analg.

[7] McCallum L, Jeemon P, Hastie CE, Patel RK, Williamson C, Redzuan AM (2013). Serum chloride is an independent predictor of mortality in hypertensive patients. Hypertension.

[8] Bei HZ, You SJ, Zheng D, Zhong CK, Du HP, Zhang Y (2017). Prognostic role of hypochloremia in acute ischemic stroke patients. Acta Neurol Scand.

[9] Hoorn EJ, Zietse R (2013). Hyponatremia and mortality: moving beyond associations. Am J Kidney Dis.

[10] Hu J, Wang Y, Geng X, Chen R, Zhang P, Lin J (2017). Dysnatremia is an Independent Indicator of Mortality in Hospitalized Patients. Med Sci Monit.

[11] Neithercut WD, Spooner RJ (1988). Nosocomial dysnatremia. Clin Chem.

[12] Sakr Y, Rother S, Ferreira AMP, Ewald C, Dünisch P, Riedemmann N (2013). Fluctuations in serum sodium level are associated with an increased risk of death in surgical ICU patients. Crit Care Med.

[13] Nagami GT (2016). Hyperchloremia - Why and how. Nefrologia.

[14] Zhang R, Wang S, Zhang M, Cui L (2017). Hyponatremia in patients with chronic kidney disease. Hemodial Int.

[15] Chiu DY, Kalra PA, Sinha S, Green D (2016). Association of serum sodium levels with all-cause and cardiovascular mortality in chronic kidney disease: Results from a prospective observational study. Nephrology.

[16] Han SW, Tilea A, Gillespie BW, Finkelstein FO, Kiser MA, Eisele G (2015). Serum sodium levels and patient outcomes in an ambulatory clinic-based chronic kidney disease cohort. Am J Nephrol.

[17] Rhee CM, Ravel VA, Ayus JC, Sim JJ, Streja E, Mehrotra R (2016). Pre-dialysis serum sodium and mortality in a national incident hemodialysis cohort. Nephrol Dial Transplant.

[18] Chang TI, Kim YL, Kim H, Ryu GW, Kang EW, Park JT (2014). Hyponatremia as a predictor of mortality in peritoneal dialysis patients. PLoS One.

[19] Ravel VA, Streja E, Mehrotra R, Sim JJ, Harley K, Ayus JC (2017). Serum sodium and mortality in a national peritoneal dialysis cohort. Nephrol Dial Transplant.

[20] Al-Chidadi A, Nitsch D, Davenport A (2017). The Effect of Serum Sodium on Survival in Patients Treated by Peritoneal Dialysis in the United Kingdom. Perit Dial Int.

[21] Dimitriadis C, Sekercioglu N, Pipili C, Oreopoulos D, Bargman JM (2014). Hyponatremia in peritoneal dialysis: epidemiology in a single center and correlation with clinical and biochemical parameters. Perit Dial Int.

[22] Mandai S, Kanda E, Iimori S, Naito S, Noda Y, Kikuchi H (2017). Association of serum chloride level with mortality and cardiovascular events in chronic kidney disease: the CKD-ROUTE study. Clin Exp Nephrol.

[23] Mendes RS, Soares M, Valente C, Suassuna JH, Rocha E, Maccariello ER (2015). Predialysis hypernatremia is a prognostic marker in acute kidney injury in need of renal replacement therapy. J Crit Care.

[24] Kidney Disease: Improving Global Outcomes (KDIGO), Acute Kidney Injury Work Group (2012). KDIGO Clinical Practice Guideline for Acute Kidney Injury. Kidney Int Suppl.

[25] Soto A, Rodríguez V, Escudero E, Hurtado A (2004). Evaluation of individual risk and mortality related factors in acute renal failure. Nefrologia.

[26] Chernick MR (2008). Bootstrap Methods: A Guide for Practitioners and Researchers.

[27] Rothman KJ, Greenland S, Lash TL (2008). Modern Epidemiology.

[28] Darmon M, Diconne E, Souweine B, Ruckly S, Adrie C, Azoulay E (2013). Prognostic consequences of borderline dysnatremia: pay attention to minimal serum sodium change. Crit Care.

[29] Stelfox HT, Ahmed SB, Khandwala F, Zygun D, Shahpori R, Laupland K (2008). The epidemiology of intensive care unit-acquired hyponatraemia and hypernatraemia in medical-surgical intensive care units. Crit Care.

[30] Akong-Moore K, Chow OA, von Köckritz-Blickwede M, Nizet V (2012). Influences of chloride and hypochlorite on neutrophil extracellular trap formation. PLoS One.

[31] Aiken ML, Painter RG, Zhou Y, Wang G (2012). Chloride transport in functionally active phagosomes isolated from Human neutrophils. Free Radic Biol Med.

[32] Sun YT, Shieh CC, Delpire E, Shen MR (2012). K+-Cl- cotransport mediates the bactericidal activity of neutrophils by regulating NADPH oxidase activation. J Physiol.

[33] Kaplan LJ, Kellum JA (2004). Initial pH, base deficit, lactate, anion gap, strong ion difference, and strong ion gap predict outcome from major vascular injury. Crit Care Med.

[34] Li H, Sun S, Yap JQ, Chen J, Qian Q (2016). 0: 9% saline is neither normal nor physiological. J Zhejiang Univ Sci B.

[35] Kellum JA, Song M, Li J (2004). Lactic and hydrochloric acids induce different patterns of inflammatory response in LPS-stimulated RAW 264: 7 cells. Am J Physiol Regul Integr Comp Physiol.

[36] Kellum JA, Song M, Almasri E (2006). Hyperchloremic acidosis increases circulating inflammatory molecules in experimental sepsis. Chest.

[37] Funk GC, Lindner G, Druml W, Metnitz B, Schwarz C, Bauer P (2010). Incidence and prognosis of dysnatremias present on ICU admission. Intensive Care Med.

[38] Rothman KJ, Gallacher JE, Hatch EE (2013). Why representativeness should be avoided. Int J Epidemiol.

